# Community health workers at the dawn of a new era: 9. CHWs’ relationships with the health system and communities

**DOI:** 10.1186/s12961-021-00756-4

**Published:** 2021-10-12

**Authors:** Karen LeBan, Maryse Kok, Henry B. Perry

**Affiliations:** 1 Independent Consultant, Washington, DC USA; 2 Department of Global Health, KIT Royal Tropical Institute, Amsterdam, The Netherlands; 3 Department of International Health, Health Systems Program, Johns Hopkins Bloomberg School of Public Health, Baltimore, MD USA

**Keywords:** Community health workers, Large-scale community health worker programmes, Community health, Community health system, Community engagement, Community participation

## Abstract

**Background:**

This is the ninth paper in our series, “Community Health Workers at the Dawn of a New Era”. Community health workers (CHWs) are in an intermediary position between the health system and the community. While this position provides CHWs with a good platform to improve community health, a major challenge in large-scale CHW programmes is the need for CHWs to establish and maintain beneficial relationships with both sets of actors, who may have different expectations and needs. This paper focuses on the quality of CHW relationships with actors at the local level of the national health system and with communities.

**Methods:**

The authors conducted a selective review of journal articles and the grey literature, including case study findings in the 2020 book *Health for the People: National CHW Programs from Afghanistan to Zimbabwe.* They also drew upon their experience working with CHW programmes.

**Results:**

The space where CHWs form relationships with the health system and the community has various inherent strengths and tensions that can enable or constrain the quality of these relationships. Important elements are role clarity for all actors, working referral systems, and functioning supply chains. CHWs need good interpersonal communication skills, good community engagement skills, and the opportunity to participate in community-based organizations. Communities need to have a realistic understanding of the CHW programme, to be involved in a transparent process for selecting CHWs, and to have the opportunity to participate in the CHW programme. Support and interaction between CHWs and other health workers are essential, as is positive engagement with community members, groups, and leaders.

**Conclusion:**

To be successful, large-scale CHW programmes need well-designed, effective support from the health system, productive interactions between CHWs and health system staff, and support and engagement of the community. This requires health sector leadership from national to local levels, support from local government, and partnerships with community organizations. Large-scale CHW programmes should be designed to enable local flexibility in adjusting to the local community context.

**Table:** 

Key messages box 1: Summary	
Key findings	
• A major challenge in large-scale community health worker (CHW) programmes is that CHWs need to establish and maintain beneficial relationships with the actors in the national health system as well as with actors in the community, and navigate these dynamic relationships over time. • For CHWs to be optimally effective, they need to be embedded in the community as members who are well known, trusted, and appreciated by community leaders, community members, and influential local groups.	
Key implications	
• CHWs need a clearly defined role that is well understood and respected, and they need to be provided with functional supports from the health system and from the community. • Community engagement in CHW programmes is a process that requires leadership at all levels in the CHW programme as well as support from the health sector, local government, and community organizations.	

## Background

When health systems are weak and resources are scarce, CHW programmes are often created as add-ons intended to increase coverage or address unmet health needs, and consequently that are not adequately integrated into the broader health system [1]. WHO defines a health system as “all the activities whose primary purpose is to promote, restore, or maintain health” [2]. WHO continues:

This…does not imply any degree of integration, nor that anyone is in overall charge of the activities that compose it. In this sense, every country has a health system, however fragmented it may be among different organizations or however unsystematically it may seem to operate. Integration and oversight do not determine the system, but they may greatly influence how well it performs. [2]

Over the past decades, various examples have been documented of how multiple, nonintegrated (and often vertical) CHW programmes, established with the best intentions, contributed to fragmented health systems that underperform [3]. This has led to the recognition that there is a need for government policy alignment across all CHW programmes, supported by a national community health strategy, with harmonization at the community level [4].

Health systems are complex and have many different interconnected components that are dynamic. CHW programmes that are to be integrated within these complex systems should be designed with the dynamic and adaptive nature of the systems in mind. To achieve Universal Health Coverage, a systems perspective should be employed to widen the focus from a cadre of CHWs to the community health system as a whole and how it interfaces with the community and with the rest of the national health system [5]. CHWs work at the interface between communities and the local health system, with varying degrees of integration into the national health system. Whatever the case, there is a need for well-defined, supportive relationships between CHWs and actors in the national health system in terms of training, supervision, supply of essential commodities and job aides, and referral pathways to enhance CHW performance and legitimize their role at the community level and with the national health system [6].

CHWs, by definition, are embedded in, drawn from, or at least work in the community. They are therefore in a good position to promote health in ways that reflect the political, environmental, social, and cultural realities of the community, partly through facilitation of broader community participation. While the interface position provides CHWs a good platform to improve community health, a major challenge in large-scale CHW programmes is that CHWs need to establish and maintain beneficial relationships with the actors in the national health system (requiring integration) as well as with actors in the community system (requiring embedment in the community). CHWs are accountable to both sides [5, 7]. CHWs navigate these multiple relationships, which are dynamic and context-dependent.

The authors used a community health system framework (as described in Paper 1 of this supplement [8]) to look at CHW relational interactions. A community health system is the set of local actors, relationships, and processes engaged in producing, advocating for, and supporting health in communities and households, outside of, but existing in relationship to, health facilities [5].

## Methods

The authors conducted a selective review of peer-reviewed and grey literature, and drew on their wide-ranging experiences with large-scale CHW programmes. They summarized lessons from large, well-established CHW programmes described in case studies from the 2020 book *Health for the People: National CHW Programs from Afghanistan to Zimbabwe* [9], and Chapters 12 and 13 from the 2014 book, *Developing and Strengthening Community Health Worker Programs at Scale: A Reference Guide and Case Studies for Program Managers and Policy Makers* [1, 3, 10]. The 29 case studies constitute almost all of the largest and most well-established national CHW programmes throughout the world. For this paper, the authors use the following definition of community: a social group comprising kin and nonkin social networks that share a sense of connectedness—through shared values, common interests, and/or adherence to norms of reciprocity—and which perceives itself as distinct in some respect from the larger society in which it resides [11]. The authors incorporate a social-ecological lens to look at multiple levels of factors influencing CHW relationships at the organizational (health system) level, the community level, the interpersonal level, and the intrapersonal level [12]. Ecological models help us understand how people, in this case CHWs, interact with their environments as they provide services [13]. Although there are a multitude of challenges that CHWs face in building trusting relationships with the health system and community, based on the literature review, the authors chose three key chronic challenges faced by CHWs in the health system space along with three in the community space for further analysis because of their importance and because they have not been emphasized in the other papers in this series.

## Results

Our findings are organized in three categories that can help health actors understand the unique bridging role CHWs play in advancing community health. First, we present a description of the “interface landscape” at the boundary between the distinct health system and community social spheres that is the operational context of the unique CHW bridging role. Second, we provide an understanding of some key challenges CHWs face in playing this role in the health system and community spheres. Finally, we give a range of response strategies that governments have employed to address these CHW bridging role challenges and to foster functional linkages and partnerships. Three widespread challenges CHWs face in playing this role in the health system include respect from higher-level health workers, facilitation of referrals, and functioning supply chains. Three particular challenges related to the development of supportive relationships between the CHW and communities include developing a healthy, trusting relationship with community actors, involving the community in the selection of CHWs, and involving the community in the CHW’s work.

### The interface landscape

Here we provide examples of enablers of and barriers to the CHWs’ vital bridging role and their interconnectedness. The space where CHWs form relationships has various strengths and tensions that need to be taken into consideration. Social interactions pervade daily life and create an abundance of social experiences. Researchers have found that people’s willingness to trust others is substantially higher after positive social experiences than after negative social experiences [14]. National health systems and community health systems are complex and consist of layers within which social interactions occur.

At the organizational (health-system) level, the quality of the relationship between the local health staff and CHWs has a strong influence on the community’s perceptions of CHWs. In Thailand, for example, these perceptions were found to be quite positive because of the partnership approach developed by the local health system and CHWs [12], while in Luwero District, Uganda, they were found to be quite negative because of unmet expectations of marginalized communities for healthcare [15].

There may also be more than one CHW serving the same catchment area in a community but with each complementing the role of the other(s). For example, in Ethiopia, besides health extension workers, there are part-time Women’s Development Army volunteers [16]. In Niger the *relais* volunteers assist the *agents de santé communautaire* [17]. And in Bangladesh there may be government- and nongovernmental organization (NGO)-sponsored CHWs working in the same community [18]. These "dual-cadre" CHW programmes often assign fewer households to each CHW, allowing for more frequent contact with community members and more time to establish trusting relationships with household members [17]. For example, volunteer polio-specific community mobilizers in India were able spend more time with polio-resistant households than could the CHWs (accredited social health activists, or ASHAs), who had many more duties [19]. Different countries choose different methods of utilizing CHWs: one full-time CHW who does multiple tasks versus part-time CHWs with more specialized tasks.

Community-level factors are context-specific and are influenced by local histories, economic and political systems, power dynamics, and sociocultural norms. Relationships are based on networking and reciprocity, and rely on trust and acceptability [5]. The challenges CHWs face in providing quality health services to clients are more pronounced in communities that have internal conflict, low levels of literacy, limited health information, distrust of the health system, strong traditional beliefs, and/or poor access to national health services [9]. CHW programmes often need to address gender discrimination and cultural sensitivities around gender existing in communities [9]. Supportive relationships between community groups, the CHW, and the local health system can positively affect behaviour change and health service utilization [20].

Potential sources of support for CHWs in the community include public sector and civil society entities such as committees, groups, and various community leaders. Examples of community groups and leaders who may or may not support CHWs include faith-based groups, self-help or mutual aid groups, schools, agricultural cooperatives, political and cultural leaders, traditional healers, traditional birth attendants, and women and youth leaders. CHW and health sector engagement with communities may take many forms, including working with both formal and informal community-based organizations that are dedicated to health, neighbourhood concerns, or other development purposes [21]. In some countries, the tribal chief or village headman represents the lowest tier of government within the community. In other countries, the traditional leadership structure may be powerful but not tied to the government. These institutions have the power to enable and support CHWs and their health activities or undermine them [20].

At the interpersonal level, CHWs can develop peer relationships with their clients through frequent home visits, building up trust over time. For example, in South Africa, CHW–client peer relationships were developed within the home-visit setting and were strengthened when CHWs provided clients with even further care and support [22]. Village health volunteers (VHVs) in Thailand were able to use their peer-status relationship with clients to provide tailored support in ways that doctors and public health officials guided by medical treatment protocols could not. A strong mutual-support peer network between CHWs could also support improved community relations, as VHVs helped each other improve problem-solving skills [12].

Intrapersonal-level factors that can positively influence CHWs’ relationships and performance, besides knowledge and skills, include exhibiting a “service mind” and natural helper characteristics, as is common among CHWs in Thailand [12]. In the Lao People’s Democratic Republic, selection of CHWs is based on a candidate’s track record of cooperation with and aid for residents [23].

CHWs need to perform a very delicate balancing act in order to function effectively in both the health system and community spheres. Addressing issues of power relations, developing trust with the community, and understanding the political, social, and economic contexts in which initiatives are supported is imperative [24]. Clarity is needed on the roles of the CHWs, the roles of CHW supervisors, and the roles of village-level health committees [25]. CHWs need skills to successfully manage their relationships with community members and with health professionals [26].

For example, during the initial phase of the Ebola outbreak in Guinea, Liberia, and Sierra Leone, there was a sharp decline in maternal, neonatal, and child health services, resulting in mistrust of CHWs by community members. When CHWs received clear directives to restart case management services, when they were trained on the “no touch policy”, and when they were provided with drugs, service provision rebounded and the CHW–community relationship improved [27]. Eventually, CHWs were also able to carry out Ebola-related activities better than outsiders [27]. CHW activities included working with community leaders and going house-to-house to provide accurate information. They worked with the national health system to search for active cases and contacts. They also helped local religious leaders to reduce transmission during funerals and burials [28].

### CHWs’ relationships with the health system

CHWs are not a panacea for weak health systems, and they require well-structured support from the health system in order to be fully effective [29]. As we discuss elsewhere in this series of papers, CHWs need a clear role definition with well-defined tasks [30], adequate financial and nonfinancial incentives [31], proper initial and continuous training [32], and adequate supervision [33]. National-, district-, or local-level health systems are responsible for providing CHWs with supervision, for receiving clients referred by CHWs, and for supplying CHWs with the required materials to conduct their service extension roles [34]. These supporting functions are mostly performed by health staff working at local-level health facilities to which CHWs are linked, as shown for 27 countries in Appendix Table 2. This table shows the local health system linkage with CHWs and the average size of the catchment area for each CHW [9].

For example, in Brazil, four community health agents work as part of a local family healthcare team comprising a doctor, nurse, auxiliary nurse, dentist, and dental hygienist [35]. They are in almost daily contact with the rest of the team, leading to closely integrated functioning. In Nepal, nine or more female community health volunteers (FCHVs) work together out of a sub-health post where female maternal and child health workers and male village health workers (VHWs) are also based [36]. In Bangladesh, the NGO BRAC’s CHWs (*shasthya shebikas*) mobilize community members to attend satellite clinics operated by the ministry of health (MOH) for immunizations, antenatal care, and family planning. They also coordinate closely with the government’s tuberculosis (TB) programme by collecting sputum specimens from symptomatic patients and sending them for testing at the district TB laboratory. For those who test positive, the government provides these CHWs with the medicines to provide directly observed treatment [37, 38]. These types of direct interactions of CHWs with the formal health system can strengthen CHWs’ feelings of connectedness by serving the same goal, yielding better performance [7].

### Challenges CHWs face when interacting with the national health system

Though numerous challenges exist, we highlight three particular chronic and widespread challenges related to establishing productive relationships between the CHW and the national health system: (1) level of respect for CHWs from higher-level health workers, (2) facilitation of referrals, and (3) functioning of supply chains.

Several common challenges have been documented in Ethiopia, Malawi, Mozambique, and Kenya by Kok et al. [7]. Issues of supervision are widespread. Regarding supervision, Kok and her colleagues identified (1) disparities in salary between the supervisor and CHW (which CHWs felt were unjust), (2) the age disparity that exists when young supervisors supervise older CHWs (which led to feelings among CHWs of being disrespected), (3) the lack of regularity of supervision (which led to feelings of CHWs not being supported by the health system), and (4) the lack or poor quality of attention given by supervisors because of their high workload and/or inadequate training (which led to CHWs feeling that they were not adequately supported by the health system). Another issue they encountered was the difference in incentives provided to CHWs by NGO-run programmes compared to those provided by MOH-run programmes, leading to feelings of unfairness among CHWs working in MOH programmes. Finally, Kok and her colleagues noted that unclear roles and responsibilities of CHWs lead to doubts among community members and higher-level health workers regarding CHW competencies, resulting in disrespect toward CHWs [7]. Issues related to suboptimal supervision, remuneration and incentives, training, and lack of clarity on CHWs’ roles and tasks are addressed in greater depth elsewhere in this series [30–33].

### Level of respect for CHWs from higher-level health workers

**Table:** 

**Key message box 2**	
The roles of CHWs and their rationale need to be made clear to other cadres of health workers in the health system so as not to undermine beneficial relationships between the CHW, national health system and communities.	

The most frequently cited barrier to “CHW programme integration” in a systematic review of scale-up and sustainability of CHW programmes was that CHWs were not respected or integrated into the hierarchy of the health system [39]. Government ownership of the CHW programme and government affirmation of CHWs as a valuable part of the workforce are likely to facilitate a more coordinated approach and result in more supportive relationships. In Cambodia, where government ownership is minimal, CHWs expressed the desire to have more involvement from the health system, such as endorsing their health promotion sessions, supplying them with a uniform and identification card, and sponsoring media campaigns that reinforce the messages the CHWs are promoting [40].

Relationships between CHWs and higher-level health workers provide legitimacy to the CHW, as perceived by the community and the CHW [41]. However, where such support is not provided or where the health facility cannot provide quality services to patients referred by a CHW, the community’s trust in and respect for CHWs can be undermined, and this can negatively influence CHW service utilization and community health [7, 42]. India’s ASHAs reported that the legitimacy provided by the national health system was important to them, but being responsive and available to community members was also important for their legitimacy and credibility as they established strong local networks and accumulated relational social capital with community members [43]. A study in Malawi found that unmet or unrealistic expectations as well as poor communication led to poor relationships between health workers interacting with CHWs and negatively influenced CHW performance [44].

Another systematic review on integration of CHW programmes into health systems, covering Brazil, Ethiopia, India, and Pakistan, found that CHWs sometimes reported feeling a lack of respect from health staff with whom they interact and in the way the staff talk about CHWs with their patients [42]. Health professionals may also disagree with decisions on task-shifting to CHWs, as CHWs take on functions that in the past were performed only by nurses or doctors—particularly some elements of curative care [45]. Although many doctors and nurses are highly supportive of strong CHW programmes, some (and some of their professional associations) nonetheless oppose task-shifting to CHWs, and others doubt CHWs’ competencies to perform their newly defined tasks [7, 42].

Health professionals, particularly physicians providing curative care at higher levels in the health system, may be unaware of the valuable role of CHWs in promoting healthy household behaviours and care-seeking for preventive services. The disrespect that some CHWs experience from health professionals may be reinforced by disparities in gender, socioeconomic status, and education, all of which can be aggravated by paternalistic and hierarchical attitudes [42]. It is of utmost importance that the roles of CHWs and their rationale be made clear to other cadres of health workers in the health system. And, of course, CHWs should be adequately trained and supported to perform their tasks [46]. If higher-level healthcare staff do not have a clear understanding of the CHW’s role, and if they believe CHWs to be inadequately selected, trained, and supervised (and therefore not suitably competent or motivated to carry out their tasks), of course they are not going to be overly enthusiastic about providing CHWs with support [25].

In some settings, CHWs resent being treated as “just another pair of hands” at the beck and call of more highly trained health workers. Some health professionals at local health facilities have sought to co-opt CHWs to become assistants for their own work within the facility and minimize their community roles [43]. Health facility-based staff in Mozambique and Zambia reported that staff deficits and poor work conditions caused heavier workloads for staff on duty, the closure of some services, and conflicts with patients, necessitating task-shifting of duties to CHWs to perform at the health facility [47]. This also occurred in Nigeria [48]. This is most unfortunate, since the greatest value of CHWs lies in reaching out beyond health facilities to people with services and health information that they would probably not obtain otherwise [49, 50].

### Facilitation of referrals

**Table:** 

**Key message box 3**	
If a CHW refers a patient to a health facility and the patient is received in a way that shows respect for the CHW and that provides the patient with quality care, then this is a sign of respect and validation of the CHW’s work.	

In many settings where access to health services is limited, especially in isolated rural areas, community members seek advice or care from CHWs when an illness arises, regardless of what training the CHW may or may not have had. In some programmes (e.g., the ASHA programme in India), special incentives and rewards for both clients and CHWs are provided when CHWs refer a patient for childbirth at a facility [51]. However, more often, CHWs receive no such benefit but instead spend time and often their own finances in accompanying clients from their community when an emergency arises [52].

Optimal referral systems require training of CHWs about what kinds of conditions require referral (such as mothers and children with danger signs of serious illness) and which conditions do not (such as cough and cold in children without signs of rapid/difficulty breathing or chest in-drawing). Having formal referral guidelines, such as protocols and referral slips, can help to make the link between CHWs and health facilities more effective. Good communication links between CHWs, supervisors, and health facility staff, along with active community engagement, can facilitate better use of referral services [53]. Mobile phones have opened up new opportunities for linking clients with higher levels of care [54].

However, when clients who have been referred by a CHW arrive at a referral facility and receive disrespectful or, delayed or inadequate care, the legitimacy and credibility of the CHW as perceived by the client and his/her family is diminished. A systematic review across 42 low- and middle-income countries documented a broad range of negative maternal healthcare provider attitudes and behaviours (such as verbal abuse, rudeness, and neglect) affecting patient well-being, satisfaction with care, and care-seeking (especially for antenatal care, facility-based delivery, and postnatal care). Reported negative patient interactions far outweighed positive ones [55]. In South Africa, while some CHWs described a trusting relationship with clinic staff, others reported that their credibility in the eyes of the community was undermined when referrals were not accepted by clinic staff or when clinic staff appeared to question the CHW’s competency or trustworthiness [56]. In one study based on focus group discussions with CHWs in Uganda [57], it was reported that “[w]orking referral systems, in particular, were seen as an important sign of respect and validation of a CHW’s work, whereas CHWs felt that referrals that were rejected or ignored undermined their relationships with the community and the value of their work.” A well-functioning referral system may serve as a motivating factor for CHWs by guaranteeing a continuum of care for the client, which can boost the relationship of CHWs with community members [53].

### Functioning supply chains

**Table:** 

**Key message box 4**	
Supply chain challenges should be anticipated and addressed proactively to ensure continuity of care provided by CHWs and to not jeopardize CHW credibility.	

A common problem encountered by CHWs in large-scale programmes has been the inability to resupply medicines and other commodities when they are needed. In fact, lack of supplies was tied with lack of financing as the most frequently cited challenge that CHWs programmes face according to the recently published compendium of national CHW programmes [9]. A recent systematic review reported that among 48 studies from sub-Saharan Africa that assessed this problem, 48% of CHWs reported that they had experienced stock-outs [58]. Two recent reports further highlight this problem. A study from Malawi found that only 29% of health surveillance assistants had the family planning methods they were supposed to have in order meet their clients’ needs [59]. In Kenya, only 6% of CHWs had all of the job aids and tools specified in the national guidelines for their maternal health activities [60].

These stock-outs are not unique to CHWs, but also affect the local health facilities to which they are attached. Interviews with key informants [58] revealed that the main causes were budgetary constraints and difficulties that CHWs may have in reaching the resupply point to replenish their stocks. Sometimes the logistics system was unable to accurately estimate supply needs. In addition, when health facilities did not receive the supplies they needed for their own services, they may have prioritized their own needs over those of CHWs who come to the facility for resupply. When stock-outs occur, CHWs are obviously unable to complete their tasks to care for patients in the community. CHWs can then lose credibility in the eyes of community members [7], jeopardizing their ongoing relationships and performance. The CHW may even be accused of stealing or selling supplies.

The importance of a functioning supply system for CHW programming was convincingly demonstrated in a recent study of the effectiveness of CHWs in reducing child mortality in Tanzania [61]. In this randomized trial, 1–59-month mortality during the first two years of the study declined by 15%, but during the final two years of the study, as a result of logistic system lapses, it increased back to baseline levels.

Even when supplies are available at the local health facility, CHWs can still face challenges in obtaining them. In Malawi and Rwanda, CHWs reported that they needed to pay out of pocket from their own money to collect drugs and other supplies [62, 63], as they had to pay for transport to reach the local health facility. These types of challenges should be anticipated and addressed proactively [29].

### Strategies to develop functional relationships between CHWs and the health system

**Table:** 

**Key message box 5**	
Integration of CHW programs into the health system, shaped by positive interactions among various actors, engenders respectful collaboration and communication between CHWs and health professionals, leading to acceptability and credibility of the CHW program.	

In their systematic review of reviews of CHW programmes that have been published in the scientific literature, Scott et al. [64] concluded that integration of CHW programmes into the national health system was a key enabler of improved CHW programme performance because it can help bolster CHW programmes during times of political upheaval, of loss external donor funding, and of reduced prioritization by the MOH. It also fosters respectful collaboration and communication between CHWs and higher-level staff, leading to acceptability and credibility of the CHW programme within the health system, trust, and beneficial relationships between actors. Integration that fosters respectful collaboration and communication between CHWs and MOH staff can enable the health system to benefit from the unique, practical knowledge that CHWs have.

In a review of national CHW programmes in Brazil, Ethiopia, India, and Pakistan [42], the authors found variable levels of integration of CHW programmes into health system elements (e.g., governance, financing, and service delivery). They concluded that policy-makers should design their national CHW programme scale-up strategy based on their own contextual factors [42]. In Thailand, for example, several factors led to stronger relationships between the VHVs and the health system, including staff appreciation of the VHVs’ contributions, regular meetings between health staff and VHVs, involvement of VHVs in planning and implementation of health programmes, and encouragement by higher-level health officials to seek recognition for VHVs [12].

In South Africa, the ward-based community outreach teams consist of a group of generalist CHWs led by a nurse. However, the team, because of its broad responsibilities for maternal and child health, HIV, TB, noncommunicable diseases, and environmental health, is also linked to specialist technical supervisors who can support them [48]. India’s now defunct Village Health Guides programme is a good example of the problems that can arise when primary healthcare (PHC) centres are ill-equipped to support CHWs [65, 66].

The engagement of the private sector to support CHW programmes is another strategy that some countries are using. Outsourcing the management of district health systems to private contractors, most notably NGOs, is one option. In Afghanistan, the government has contracted NGOs to recruit, train, and support CHWs, lessening the burden on an already weak health system [67]. Cambodia is also a case in point. The programme management, training of CHWs, and delivery of services is provided by nongovernmental agencies with donor funding. This has led, however, to ill-defined ownership of the programmes and a lack of government accountability, leadership, and management [40].

### CHWs’ relationships with communities

**Table:** 

**Key message box 6**	
CHWs’ supportive relationships with community leaders, members and community groups are important enablers of CHW retention, motivation, performance, and accountability.	

Just as good relationships are desirable between CHWs and the health system, the CHWs’ relationships with community leaders, members, and community groups are important enablers of CHW support, retention, motivation, performance, and accountability, and ultimately of the acceptability and uptake of their health-related work [64, 68]. Evidence indicates that CHWs can engage and mobilize the community to improve a range of health issues [68]. However, lack of community support or perceived low value of the CHW by community members are common barriers to scaling up and sustaining CHW programmes [39].

Appendix Table 3 provides an overview of the role of the community in CHW selection, programme implementation, supervision, and performance evaluation in 29 large-scale CHW programmes. This information is abstracted from *Health for the People: National Community Health Worker Programs from Afghanistan to Zimbabwe* [9]. According to the 2018 WHO guideline on optimizing CHW programmes, a range of community engagement strategies have been found to have a beneficial impact on CHW performance outcomes, including strategies to build trust in the CHW, to promote community awareness about the capabilities of CHWs and the limits of their capabilities, and to build support for and create a sense of ownership of CHW programmes [68, 69]. Paper 7 in this series, which focuses on supervision of CHWs, also provides additional insights into contributions that communities can make in the supervision of CHWs [33]. However, detailed information on these issues remains limited [70].

### Challenges CHWs face when interacting with the community

**Table:** 

**Key message box 7**	
Time and resources are necessary to ensure that communities have a clear understanding and realistic expectations of the CHW program during its inception and as it evolves over time.	

Though numerous challenges exist, here we highlight three particular ones related to the development of supportive relationships between the CHW and community actors: (1) defining a healthy, trusting relationship with community actors, (2) involving the community in the selection of CHWs, and (3) involving the community in the work of CHWs [68, 69]. Constraints to beneficial relationships between CHWs and the community can be both external and internal [71]. External obstacles include pressure from the donors and technical advisors to achieve quick results (thereby bypassing the slower social processes that can be required for establishing stronger ties between the CHW and the community), co-optation of community participation by the government (e.g., using the political system as a form of social control), and the creation of processes for selection of CHWs that favour the selection of the politically well-connected. Further, governments and development groups may favour investment in interventions with easily measurable indicators and may underinvest in the more intangible social processes and community participation that are critical to longer-term success and sustainability. Internal obstacles include conflicting local interest groups, gatekeeping by local elites, and local apathy. It is not uncommon for CHWs to work in communities with internal social conflicts [72]. A CHW can often do little to overcome these factors that are inherent in the system [71].

### Defining a healthy, trusting relationship with community actors

The importance of building beneficial relationships between CHWs and community actors can be easily overlooked when a CHW programme is being designed or when the programme is being adapted to meet new needs [73]. There are a variety of community participation approaches that run along a continuum from passive to transformative [74]. Not all large-scale CHW programmes only focus on passive information sharing. Even though programme consultation and collaboration with community leaders and local government is a key element for building community ownership of the CHW programme, local health staff often do not have the necessary time and energy to facilitate understandings that communities have a realistic understanding of the CHW programme both during the inception of the programme and as the programme evolves over time. If community leaders do not facilitate support for the CHW, such as at regular community meetings, community trust and respect towards the CHWs can be undermined [25, 75]. Government reforms and policies that support the devolution of responsibilities to local groups (for example, policies that mandate inclusion of communities in programme planning) may not exist [76]. Unrealistic expectations may contribute to poor CHW programme performance [64, 77]. If community members do not see that a CHW has something to offer them, then collaboration will be difficult. For example, while development and education activities are important, community members may also expect some curative services from CHWs [78]. Research has shown that the addition of curative services to CHWs’ roles and tasks can lead to enhanced recognition and respect from the community towards CHWs and enhanced feelings of self-fulfilment of CHWs, which positively influence CHW performance [7].

### Involving the community in selection of CHWs

**Table:** 

**Key message box 8**	
Transparent strategic selection of CHWs by community members in open and inclusive events contribute to community cooperation.	

CHWs who are not embedded in the community have a distinct disadvantage in efforts to engage the community in supporting their work. Community embeddedness can be defined by the CHW’s social connections in and knowledge of the community [79]. In one systematic review, the most frequently cited factor causing community lack of acceptability and accountability of their CHW was if the recruitment of CHWs was not from and by the community [39].

Communities generally have a role in the selection of their CHWs by helping to set selection criteria, nominating candidates, and/or through actual selection. However, community engagement in the selection of CHWs can also produce problems, as evidenced in the literature. Where the CHW selection process is not transparent to community members, suspicion of favouritism may arise that could lead to jealousy or loss of willingness of the community to cooperate. When CHW selection is managed by traditional kinship structures, this could aid community participation and intervention effectiveness within the kinship group, but can lead to exclusion of others [80]. Uganda decided to allow every kinship or neighbourhood group to select as many community-directed health workers as practical in its onchocerciasis programme [81]. In India, researchers found evidence of nepotism and favouritism in the selection of ASHAs by community leaders benefitting the already privileged [82].

Policy preferences for CHW selection need to reflect community realities. For example, in fragile settings such as in Liberia and the Democratic Republic of the Congo, it was challenging to find candidates who were literate, though the policy included literacy as a requirement [83]. In Sierra Leone and Liberia, the policies state a preference for female CHWs. However, in Sierra Leone there were more male candidates due to hesitancy on the part of women to apply (due to cultural norms), while in the Democratic Republic of the Congo there were more female CHW candidates because of the many active women’s associations there [83].

When selected and employed by the government without community participation, CHWs may feel more responsible to their employer than to the community [84, 85]. Government-employed CHWs may also spend more time supporting health centre services if there is a shortage of qualified personnel there, leaving less time to respond appropriately to the community’s needs [86]. Paper 6 in this series, on recruitment, training, and continuing education, provides additional perspectives on the role of the community in the selection of CHWs [87].

### Involving the community in the work of the CHW

**Table:** 

**Key message box 9**	
While CHW representation in community-based organizations (CBOs) can lend voice to communities, enhance community ownership and support greater community participation, these CBOs are generally weak and often have problems with community participation, member selection, and establishing supportive relationships with the health system.	

The processes for involving the community in working together with its CHW to respond to locally felt needs are not widespread [9]. The Updated CHW Functionality Matrix for Optimizing Community Health Programs considers the community highly functional if the community provides input during the planning of the CHW programme, monitors CHW activities, and provides feedback to the health system [88]. While CHW representation in CBOs, community health committees (CHCs) or village health committtees (VHCs) can lend voice to communities, enhance community ownership, and support greater community participation. These structures are generally weak and often have functionality problems such as lack of community participation, biased member selection, and weak relationships with the health system [89]. A study in Kenya found that CHCs were disconnected from other actors in the health sector and that they hardly played a role in CHW support and community participation [90]. A study in India found that village health and sanitation committees (VHSCs) performed few of their specified functions for decentralized planning and actions, and concluded that they need education, mobilization, and monitoring [91]. Another study of the VHSCs in India found that these committees can also engender social transformation by providing social space for women to interact with men, but the process required specialized outside facilitation [92]. In Malawi, insufficient clarity on the roles and responsibilities of the village health committees to the CHWs resulted in low community involvement [93].

### Strategies to develop functional relationships between CHWs and community actors

Building community capacity serves as a means to an end—improving health behaviors and collective action for health—and is also an end in itself [94]. A systematic review revealed that communities with more trust in, respect for, and recognition of CHWs had CHWs with higher levels of motivation, self-esteem, self-assessed performance, and adherence to guidelines [25]. CHWs play different roles in different countries, and evidence is growing about how CHWs can engage with communities in their different roles. Based on systematic reviews of evidence and its own expertise, WHO made a strong recommendation for the adoption of the following community engagement strategies in the context of CHW programmes:

- Programme consultation with community leaders
- Community participation in CHW selection
- Community monitoring of CHWs
- Community involvement in selection and priority-setting of CHW activities
- Support to community-based structures (such as CHCs)
- Involvement of community representatives in decision-making, problem-solving, planning, and budgeting processes [68, 69].

Table 1 provides examples of the importance of community engagement related to different roles a CHW may have and the ways CHWs can engage community members to participate in various health tasks, leading to stronger relationships. Each role requires effective counselling. Counselling is not simply a skill that can be taught, but is also dependent on a relationship of trust between the CHW and client, which is built up over time as CHWs accrue social capital in the community [95].

**Table 1:** Different forms of community engagement for specific types of CHW roles [10]

CHW role	Importance of community engagement	Ways the community can participate
Health promoter/health educator, including communication, counselling, and support to improve health and prevent disease	Behaviour change requires repeated, intensive contacts over a period of time, and is influenced by peer support and community norms	Participatory community or peer groups who witness visible change provide support and continuity for individual behaviour change
Healthcare provider, including treatment of common illnesses, referral to health facilities, and care and support to the chronically ill	Cultural perceptions of illness and treatment may affect prevention, treatment, and care Social ties influence health outcomes [96]	Participation in quality improvement activities Selection of specialized volunteer cadres who are patient advocates or who support referrals made by CHW
Agent of change, including support for community mobilization, empowerment, and human rights	Community buy-in and action are required to address some of the structural influences on health (e.g., power dynamics, poverty, and discrimination)	Engagement in the problem-posing and problem-solving process at community meetings can lead to collective action to change community circumstances
Health manager/enumerator, including vital event and other reporting	Communities may not want to provide vital events information to government officials, but they may be willing to provide this information to a trusted CHW	Visible community health information boards Discussion of changes in health situation at community meetings

*Health promoter/health educator* Each community may have several peer groups and community-based organizations (such as community associations) that can support the CHW and improve health outcomes. Examples of these peer groups include women’s groups, youth groups, men’s groups, groups of HIV/AIDS patients, water and hygiene groups, and breastfeeding groups. CHWs often lead these groups or are group members. The success of CHWs achieving behaviour change to prevent disease and improve health requires repeated contact between community members and CHWs over a period of time, and is aided by peer support and community norms. Ultimately, group members who witness visible benefits from their own behaviour change are able to provide the support and continuity necessary for sustained behaviour change among others.

A particularly effective approach to using CHWs to promote behaviour change for child survival that has been widely used by the international NGO community is the use of Care Groups [97]. A paid promoter (CHW) travels from village to village to meet with Care Groups, which consist of 10 volunteer CHWs, each of whom is responsible for approximately 10 households. The Care Group meets once or twice a month for about two hours to learn and discuss a relevant health message. Then, the volunteer CHW shares the message with the households for which she is responsible. The Care Group volunteers meet together as a Care Group 2–4 weeks later to discuss their experiences (and also, in many projects, to report vital events). Then the cycle continues [97].

*Healthcare provider* Cultural perceptions of illness and treatment may undermine prevention, treatment, and care options. Community support groups can help bridge local belief patterns and specific local terminology with modern biomedical knowledge. There are influential people in each community such as community leaders, chiefs, traditional medicine practitioners/healers, religious leaders, teachers, and business people who are recognized and respected in their communities. They can accompany CHWs on household visits to lend credibility to their behaviour change messaging, counsel families on specific health issues, and provide advocacy at community meetings. Some of these individuals may become patient advocates. Others can support referrals and help with surveillance of communicable diseases, such as traditional healers referring patients with HIV and TB to health facilities in Mozambique [98].

*Change agent *Within the community, the social environment has an indirect but powerful effect on the practice of personal behaviours that promote good health. Among marginalized populations in particular, isolation, poverty, low self-esteem, discrimination, lack of social support, nomadic living, language barriers, and discriminatory power barriers are common. These factors may limit knowledge, bias attitudes, and prevent the practice of lifesaving, preventive, home and care-seeking practices [74]. In these situations, CHW tasks can be complemented by timely messaging that is delivered through multiple channels (such as religious sermons, street theatre and radio, government broadcasts, social media, and/or community dialogue) [99].

*Health manager/enumerator *CHWs can provide an important role in collecting household data, vital events, and disease prevalence information from the community that can be used to involve community members in problem-solving for health. This information can also be used by the national health system to focus on emerging health issues. Visible community scoreboards can be created to provide community access to this information.

Community-based organizations, village health committees, health facility management committees, and discussion forums can lend voice to communities and support greater participation in the work of CHWs [76]. CHWs in some villages in Kenya reported that they had a close relationship with their CHC, creating an environment of mutual support. Because of the repeated, familiar interaction between the CHW and the household, CHWs were witness to instances of substance abuse, partner violence, environmental hazards, and child neglect (among other community problems) requiring social support beyond their training. Community-based organizations can help to address issues such as these. They can also organize community health action days and dialogue days with the CHW and other influential members of the community [26]. CHWs can collect information from households on vital events and disease prevalence and then engage the community with this information, thereby enhancing supportive community relationships as well as CHW motivation [100, 101].

In Assam State, India, one ASHA facilitator was hired for every 10 ASHA workers to provide support in holding VHSC meetings, counselling families, accompanying ASHAs on visits to a home with a newborn, and supporting immunization and antenatal care services [102]. In Guinea, Liberia, and Sierra Leone, the CHCs were a natural platform for the establishment of Ebola task forces and subsequent coordination activities with CHWs [27]. In Brazil, municipal health councils, which provide a major part of the funding for CHWs, took an active role in the local PHC programme, of which community health agents are a vital part [35].

There are several examples of CHW programmes in which CHWs have strong connections with the community that facilitate their work. The FCHVs in Nepal are selected by mothers’ groups. The FCHVs facilitate their meetings and the mothers’ groups assist the FCHVs with their work [36]. In the BRAC CHW programme in Bangladesh, *shasthya shebikas* are members of a women’s group (called a voluntary organization) which is a micro-credit group [38]. In Rwanda, CHWs work with community hygiene clubs and also facilitate parents’ “evening forums”, where parents bolster CHW promotion efforts around sanitation, behaviour change, and broader health issues [103]. The CHWs in India (auxiliary nurse midwives, ASHAs, and anganwadi workers) are all members of VHSCs, and the ASHA is the convener of the committee [104]. In Afghanistan, CHW Days are held in communities throughout the country to express appreciation to CHWs for their services, fostering stronger relationships [67].

CHW performance could benefit from joint ownership and design of CHW programmes by the health sector and communities. Researchers have recognized that, while joint ownership begins at the national level, it is expressed operationally at the community level. National-level dialogue could produce a framework for how collaboration might work, and local leaders and stakeholders could adapt and adjust normative guidance from the national level to meet the needs of local conditions [11]. One approach focuses on identifying key functional components of effective support and then identifying feasible local approaches to providing them [105].

An effective CHW programme can serve as a catalyst for community engagement, but this is sustainable only as long as the relevant actors remain committed and the sociopolitical and economic environments remain conducive to the process [106]. Recently, some have argued that mechanisms to engage citizens, especially through CHWs, are essential for high-quality interventions as well as for inclusive disaster response and preparedness programmes, such as those developed for COVID-19 [107]. Substantial and time-consuming investments are needed to secure the participation of the community at programme inception and for programme maintenance [108].

### Limitations

The topic of this paper is a vast one, and all the articles that address these issues are not readily identified because many are focused primarily on other aspects of CHW programmes. The literature that we cite here is probably just the “tip of the iceberg”, but it is at least a beginning that others can build on. In spite of these limitations, we hope that the approach used here and the articles we cite can serve as a beginning point to investigate these issues in greater depth, to summarize more systematically the current state of knowledge, and to propose specific research questions to advance our knowledge of this important topic.

## Conclusion

Community-based health programming focused on the promotion of healthy household behaviours, access to preventive services, management of common illnesses, and referral of those in need to higher-level medical care should be the foundation of an effective health system. The CHW is in an intermediary position between the health system and the community. While this position can provide significant advantages in terms of advancing community health, it also requires that CHWs establish functional relationships with both sides, which have different expectations and needs. Therefore, CHWs need to have a role that is clearly understood by all parties, and CHWs need strong interpersonal communication skills. There are chronic and widespread challenges to CHWs successfully playing their vital bridging role. CHWs can be integrated into the hierarchy of the health system, with health professionals respectfully acknowledging the CHWs’ role. Functioning patient referral and logistical supply systems are critical for the effective functioning of CHW programmes. Giving the community a role in planning the CHW programme as it evolves, selecting their CHW, and supporting their CHW to help him/her be effective are also critical for the effective functioning of CHW programmes.

Policymakers, funders, and programme managers need to know what conditions support CHWs to form quality relationships. Factors at the policy, local health system, community, interpersonal, and intrapersonal levels can enable or constrain beneficial relationships between the CHW, the health system, and communities. Each CHW programme should make explicit how it expects community health services to contribute to health goals at different levels, including national health goals. This requires that programme managers understand what major disease burdens can be addressed by CHWs, which behaviours—if changed—would yield the greatest impact, what major interventions can avert death and serious morbidity, and which of these can be delivered in the community.

Community–CHW relationships are dynamic and iterative and are linked to social, political, and economic factors, reflecting that community participation is context-specific. Engaging the community is a process that requires leadership at all levels in the CHW programme, the support of the health system and local government, and partnerships with community organizations. Better documentation and research on CHW relationships with the health system and communities, as well as enablers of and barriers to effective relationships, should be conducted more systematically in order to understand which factors have the most impact on CHW programme performance. Learning from the experiences of large-scale CHW programmes, anticipating common challenges faced by these programmes, and applying these lessons within the appropriate national and subnational context will be essential if the failures of large-scale CHW programmes in the 1980s are not to be repeated and if CHW programmes are to reach their full potential.

## Data Availability

All data used are referenced appropriately and widely available.

## Appendix

See Appendix Tables 2 and 3.

**Table 2:** Local health system linkage with CHWs and CHW catchment area by country

Country	CHW cadre	Local health facility to which CHW is attached	CHW catchment areas population served
Afghanistan	Community health workers (working at health posts and in the community)	Basic and comprehensive health centres	1 male and 1 female in each health post serving a minimum of 150 households
Bangladesh government	Family welfare assistants Health assistants Community healthcare providers	Health and family welfare centres (government) Community clinics (government) NGO health centres	Maximum of 500 eligible couples 6000 people 6000 people
Bangladesh BRAC	BRAC *shasthya shebikas* BRAC *shasthya kormis*	None	450 households 4500 households
Brazil	Community health agents (5–6 per family health team), and several family health teams for each health centre	Health centres	750 people per CHA 3500 people per family health team
Ethiopia	Health extension workers (working at a health post and in the community) Women’s Development Army volunteer	Health centres	2500–4000 people 5 households
Ghana	Community health officers (at community clinics) Community health volunteers	Subdistrict clinics and health centres	5000 people 2500 people
Guatemala	CHWs (health guardian)	Health centres/health posts	2500 people
India—current	Auxiliary nurse midwives Anganwadi workers Accredited social health activists	Community health centres, sub-centres, and primary healthcare centres	2000–3000 people 1000 people 1000–2500 people
India 1997–2002	Village health guides	Data not available	Data not available
Indonesia	*Kaders*	Community health centres or sub-centres	700 people Kaders run the *posyandus*, each serving about 100 children and 10–20 families; and about 700 people in the community
Iran	*Moraghebe-salamats* based in urban health posts *Behvarzs* based in rural health posts	Comprehensive health centres	1000–3000 people
Kenya	Community health volunteers	Primary healthcare centres and community health units	500 people (100 households)
Liberia	Community health assistants	Clinics	350 people
Madagascar	*Agents communautaires* (ACs) Nutrition ACs	Basic health centres	Varies Varies
Malawi	Health surveillance assistant (HSAs)	Health centres and maternity facilities	1000 people
Mozambique	*Agents polivantes elementares* (working at health posts)	PHC centres	Between 500 and 2000 people
Myanmar	Auxiliary midwives Community health workers Malaria volunteers TB volunteers	Health centres and sub-centres	Varies Varies Varies Varies
Nepal	Female community health volunteers	Health posts (staffed by full-time paid staff)	365 people
Niger	*Agents de Santé communitaire* *Relais* volunteers	Health posts	2000 people Varies
Nigeria	Community health extension workers Voluntary village health workers	Health centres	4000 people 1000 people
Pakistan	Lady health workers	Health centres	1000 people
Rwanda	*Binôme* (male and female pair) *Animatrices de Santé Maternelle*	Health centres	2 per village 1 per village The 3 together serve 50–150 households
Sierra Leone	Community health workers	Peripheral health units	250 people in hard-to-reach areas and 1000 people in easy-to-reach areas
South Africa	Community health workers	Health centres (public and private mix)	140–250 households
Tanzania	Community health workers Volunteer community health workers	Health dispensaries and health centres	Not available Not available
Thailand	Village health volunteers	Health centres	10–20 households but varies
Uganda	Village health team members	Health centres	250 people
Zambia	Community health assistants Community-based volunteers	Health centres and health posts	3500 people Not available
Zimbabwe	Village health Workers	Health centres	100 households

Information obtained from *Health for the People: National CHW Programs Afghanistan to Zimbabwe*, 2020 [9]

**Table 3:** Community engagement with CHWs by country

Country	Role of community in:			
	CHW selection	CHW programme implementation	CHW supervision	CHW job performance evaluation
Afghanistan	Yes	Yes, women’s groups	Yes, village health committees	Yes
Bangladesh—government	No explicit role	Yes, community groups	Not stated	Not stated
Bangladesh—BRAC	Yes	Yes	Not stated	Not stated
Brazil	Yes	Not stated	Not stated	Not stated
Ethiopia	Yes	Yes, village health committees	Kebele council members	Kebele council members
Ghana	Varies	No	Yes, community health management committees	Not stated
Guatemala	Yes	Yes	No	No
India—current	Varies	Varies	Varies	Varies
India 1997–2002	Varied	No	No	No
Indonesia	Yes	Not stated	Not stated	Not stated
Iran	Yes	Not stated	Not stated	Not stated
Kenya	Yes	Yes	Varies	Varies
Liberia	Yes	Yes, community health committees	Yes, village health committees	Not stated
Madagascar	Varies	Varies	Yes, village general assembly	Not stated
Malawi	Yes	Yes	Yes, village health committees and community health management committees	Yes
Mozambique	Yes	Yes, community leaders	Yes, community health committees	Not stated
Myanmar	Varies	Varies	Not stated	Not stated
Nepal	Yes	Yes	Yes, health facility management committees	Not stated
Niger	Yes	Yes	Yes, health facility management committees	Not stated
Nigeria	Yes	Yes, ward development committees	Yes, ward development committees	Yes
Pakistan	Yes	Yes	Yes, women’s and men’s health committees	Yes
Rwanda	Yes	Yes, community clubs	No	Not stated
Sierra Leone	Yes	Yes, community groups	Yes	Yes
South Africa	Minimal	No	No	No
Tanzania	Varies	Yes	Yes	Yes
Thailand	Yes	Not stated	Not stated	Not stated
Uganda	Yes	Yes	No	No
Zambia	No	Not stated	Not stated	Not stated
Zimbabwe	Yes	Yes, community leaders	Yes, community leaders	Yes

Information obtained from *Health for the People: National CHW Programs Afghanistan to Zimbabwe*, 2020 [9]

“yes” if case study referred to this role; “no” if case study made the point that the community had no role; “not stated” if this role was not examined in case study

## Abbreviations

AIDS: Acquired immunodeficiency syndrome
ASHA: Accredited social health activist
CBO: Community-based organization
CHC: Community health committee
CHW: Community health worker
FCHV: Female community health volunteer
HIV: Human immunodeficiency virus
MOH: Ministry of Health
NGO: Nongovernmental organization
TB: Tuberculosis; VHV: Village health volunteer
VHV: Village health volunteer
VHSC: Village health and sanitation committee
